# Non-Alcoholic Fatty Liver Disease in Patients with Polycystic Ovary Syndrome: A Systematic Review, Meta-Analysis, and Meta-Regression

**DOI:** 10.3390/jcm12030856

**Published:** 2023-01-20

**Authors:** Ramiro Manzano-Nunez, Marta Santana-Dominguez, Jesus Rivera-Esteban, Clara Sabiote, Elena Sena, Juan Bañares, Frank Tacke, Juan M. Pericàs

**Affiliations:** 1 Liver Unit, Vall d’Hebron University Hospital, 08035 Barcelona, Spain; 2 Vall d’Hebron Institute for Research, 08035 Barcelona, Spain; 3 Faculty of Medicine, Universitat Autònoma de Barcelona, 08035 Barcelona, Spain; 4 Gynecology and Obstetrics Department, Hospital Clínic de Barcelona, 08036 Barcelona, Spain; 5 Department of Hepatology & Gastroenterology, Charité Universitätsmedizin Berlin, Campus Virchow-Klinikum and Campus Charité Mitte, 10117 Berlin, Germany; 6 Centros de Investigación Biomédica en Red, Enfermedades Hepáticas y Digestivas (CIBERehd), 28029 Madrid, Spain

**Keywords:** non-alcoholic fatty liver disease, metabolic dysfunction associated fatty liver disease, polycystic ovary syndrome, prevalence, risk factors

## Abstract

Background: The metabolic effects of polycystic ovary syndrome (PCOS) may increase the risk of non-alcoholic fatty liver disease (NAFLD). However, the burden of NAFLD in PCOS has not been unequivocally defined. This systematic review (SR), meta-analysis (MA) assessed NAFLD’s prevalence, and risk factors in patients with PCOS. Methods: A literature search was performed in MEDLINE, Scopus, and Scielo. First, we performed a MA of proportions to estimate the prevalence of NAFLD in PCOS. Second, we performed meta-analyses of precalculated adjusted odds ratios to examine NAFLD risk factors. Finally, we performed a meta-regression to model how the estimated prevalence changed with changes in prespecified variables. Results: We identified 817 articles from the database searches. Thirty-six were included. MA of proportions found a pooled NAFLD prevalence of 43% (95% CI, 35–52%) with high heterogeneity (I^2^ = 97.2%). BMI, waist circumference, ALT values, HOMA-IR values, free androgen index levels, hyperandrogenism, and triglycerides were associated with significantly higher risk-adjusted odds of NAFLD among patients with PCOS. Meta-regression showed that rises in NAFLD prevalence were mediated through increases in metabolic syndrome prevalence and higher levels of HOMA-IR, free androgen index, and total testosterone. Conclusion: The prevalence of NAFLD (43%) among PCOS patients is high despite their average young age, with several metabolic and PCOS-specific factors influencing its occurrence. Screening programs may aid in detecting metabolic-associated fatty liver disease and prevent its consequences. Further work is required to establish the burden of liver-related outcomes once NAFLD has progressed in the PCOS population.

## 1. Introduction

The increasing burden of non-alcoholic fatty liver disease (NAFLD), which now is estimated to affect approximately 25% of the global population [1], has become a matter of global concern as the disease comes with a higher risk of other metabolic impairments, including obesity, type 2 diabetes mellitus, and the consequent likelihood of poor cardiometabolic and liver-related outcomes [2]. The clinical challenge with NAFLD is that it can progress to non-alcoholic steatohepatitis and cirrhosis, well-known drivers of hepatic decompensations and mortality, if not identified and managed.

Polycystic ovary syndrome (PCOS) is characterized by hyperandrogenism, ovulatory dysfunction, and polycystic ovarian morphology [3]. It is closely linked with obesity, insulin resistance, abnormal glucose metabolism, dyslipidemia, and related disorders, which increase the chance of metabolic-associated abnormalities, including NAFLD. Indeed, previous studies have demonstrated an association between PCOS and NAFLD [4,5,6], and it has been postulated that the metabolic environment of PCOS patients favors the build-up of fat in the liver. Therefore, it is paramount to determine the burden of NAFLD in patients affected by PCOS, and further identify the factors mediating the relationship between NAFLD and PCOS. Understanding the link between the two disorders will advance the field of NAFLD in special populations and aid stakeholders and decision-makers in designing and implementing actions to tackle the NAFLD burden, focusing on groups at higher risk of the condition.

This study aimed to assess NAFLD’s prevalence and risk factors in patients with PCOS.

## 2. Materials and Methods

The present SR and MA was performed following the recommendations from the Cochrane Handbook for Systematic reviews of interventions, and the Preferred Reporting Items for Systematic reviews and Meta-Analyses (PRISMA) statement [7,8]. However, as the present report was expected to be based primarily on observational data, the Meta-analyses of Observational Studies in Epidemiology (MOOSE) checklist was used to report this SR and MA [9]. The MOOSE checklist is available in the Supplementary File.

To accomplish this paper’s objectives, this SR and MA answered the following question:

What is the prevalence and factors associated with NAFLD in patients with PCOS who have no concurrent viral hepatitis co-infection or significant alcohol use?

### 2.1. Eligibility Criteria

Papers were considered eligible for inclusion in this SR if they assessed the presence of NAFLD in patients with PCOS.

### 2.2. Inclusion Criteria and Exclusion Criteria

#### 2.2.1. Types of Studies

We included observational studies, including cohort, case–control studies, and case series. We also considered randomized controlled trials. These studies were included if the proportion of patients with NAFLD among PCOS patients was assessed. In addition, articles that assessed the factors associated with NAFLD were also considered eligible. Case reports were not considered eligible for inclusion.

#### 2.2.2. Participants

The participants were women with PCOS diagnosed by standard criteria [3]. Articles were included in this study if the diagnosis of PCOS was performed by any of the following: (1) National Institutes of Health criteria, (2) Rotterdam criteria, or (3) the Androgen Excess and PCOS Society criteria. These criteria share several diagnostic elements, including the combination of hyperandrogenism, ovulatory dysfunction, and polycystic ovarian morphologic features [3]. Cochrane guidelines [8] advise that reviews should be sufficiently broad to encompass the likely diversity of studies, and inclusion criteria should aim to include all relevant clinical features with which patients of interest would present. Therefore, we did not restrict the participant’s inclusion criteria to a specific set of diagnostic criteria because all of them reflect the clinical problem of interest (PCOS). Excluding studies based on the diagnostic criteria used to diagnose PCOS may result in exclusion of informative studies containing clinically relevant data from patients with the clinical problem of interest.

Studies were excluded if they included patients with either clinically relevant concomitant alcohol consumption or a history of viral hepatitis co-infection.

### 2.3. Outcomes

The prevalence of NAFLD in PCOS patients, and the factors associated (risk factors) with such prevalence were the outcomes of interest in this systematic review.

NAFLD diagnosis was based on the detection of hepatic steatosis, defined as the presence of significant steatosis demonstrated either by biopsy or a non-invasive test. For this SR, biopsy, non-invasive imaging tests, and blood biomarkers/panels were considered appropriate diagnostic methods, and thereby eligible for inclusion [10].

Non-invasive imaging tests included right upper quadrant ultrasound, computer tomographic (CT) scan, magnetic resonance imaging (MRI) techniques, or vibration-controlled transient elastography (VCTE)-based attenuation parameter (CAP) measurements. Non-invasive blood biomarkers and panels are described elsewhere [11]. If studies reported the assessment of liver steatosis by any of the methods mentioned above with their correspondent definition, then the study was considered to inform the primary outcome.

Regarding the risk factors for NAFLD in PCOS patients, we were interested in adjusted measures of association between PCOS and NAFLD. Therefore, reported measures relating to patient characteristics, relevant clinical data, and comorbidities were extracted from the included studies. The extracted risk factors had to result from a multivariable regression analysis performed in a PCOS population with NAFLD as the outcome variable and reporting adjusted odds ratios with 95% confidence intervals for the covariates of interest. We did not pre-specify risk factors of interest; instead, we extracted those available in the included studies. We collected the risk factors (adjusted odds ratios from a multivariable regression analysis) that were available in the included articles. If two studies or more reported the same factor, we considered it appropriate for meta-analysis.

### 2.4. Electronic Search Strategies

Following experts’ recommendations [12,13], we outlined a systematic search strategy of the available literature. The literature search was performed from inception to June 2022 in SCOPUS, MEDLINE (through Ovid), and Scielo. The searches were not restricted to language or geographic location. The systematic database searching was complemented by a snowball scanning of the references cited in the included studies. The structure of the search strategies in the electronic databases was informed by the main concepts of the review, combining controlled vocabulary and synonyms related to the population of interest (patients with PCOS) and the disease/condition of interest (NAFLD). The search strategies that were executed in SCOPUS, MEDLINE, and Scielo are described in detail in the Supplementary File.

We did not attempt to explore the grey literature in this systematic review.

### 2.5. Study Selection

The initial phase of the study selection process was performed blindly and independently based solely on titles and abstracts. With this purpose, the results from the search strategies were imported into Rayyan [14]. Then, two authors (RM, MSD) independently screened the titles and abstracts, and selected potential articles for inclusion based on the inclusion and exclusion criteria. Finally, articles that appeared relevant to this SR’s topic were retrieved as full text, and subsequently reviewed by two investigators (RM, MSD) who independently applied inclusion and exclusion criteria to full texts for final eligibility. We included articles in English, Spanish, French, Italian, and Chinese. In both phases (screening and full-text review), the systematic review team leader (JMP) resolved disagreements over article eligibility. For example, if the same article was marked as included by one of the investigators (RMN, MSD) and excluded by the other, then JMP made the final decision regarding its inclusion.

In cases of overlapping populations (i.e., different papers reporting data from the same population or during overlapping periods), the publication with the larger sample size or greater data granularity was selected for inclusion in the SR.

### 2.6. Data Collection

We collected the data of interest from each study into a pre-designed data collection form. The data was collected as reported in each study and included: authors, year of publication, study design, region of origin, number of patients (in comparative studies: number of PCOS and control patients), comorbidity information, and relevant lab values. We extracted and charted data only from PCOS patients. In the case of comparatives studies, the control group’s data were not considered nor extracted.

We also extracted each study’s objectives and inclusion/exclusion criteria. This information was extracted and collected in the same form as reported in each study and is available in Table S1 in the Supplementary File.

### 2.7. Risk of Bias

We used the Methodological Index for Non-Randomized Studies (MINORS) [15] tool to assess the quality and internal validity of the studies included in this SR. The explanation of how to use it is described elsewhere [15]. MINORS evaluates the methodological quality of non-randomized studies across eight methodological items in cases of observational studies without a comparison group. Four additional items are added in the case of a comparative study. Each item is scored as 0: if not reported (Red: high risk of bias); 1: reported but inadequate (Yellow: unclear risk of bias); and 2: reported and adequate (Green: low risk of bias). The results from the MINORS evaluation are available in the Supplementary File. Publication bias was not evaluated because tests to evaluate this kind of bias were created to be performed in cases of comparative data. However, there is no evidence that these tests are appropriate for meta-analyses of proportions. On the contrary, conventional methods to assess publication bias are inaccurate in this context [16], and experts recommend against performing statistical calculations to assess it when conducting meta-analyses of proportions [17].

### 2.8. Data Synthesis and Meta-Analysis

Information from each study was summarized descriptively. The prevalence (proportion) of NAFLD in PCOS was obtained by dividing the number of NAFLD cases registered in PCOS groups by the total number of patients with PCOS. We used the “metaprop_one” command [18] in Stata v.14 to conduct the meta-analyses examining the pooled NAFLD prevalence.

The “metaprop_one” command is appropriate to pool proportions in a meta-analysis of proportions to estimate the prevalence of an event of interest, such as in this SR. In brief, confidence intervals for the individual studies were calculated using exact confidence limits for a binomial proportion. Pooled event rates (pooled prevalence) were estimated through a meta-analysis of binomial data with the Freeman-Tukey double arcsine transformation of proportions [18]. The results from this analysis were presented in a forest plot presenting the study-specific proportions with 95% confidence intervals the I^2^ statistic and the overall pooled estimate (the estimated NAFLD prevalence).

Risk factors of NAFLD among patients with PCOS were meta-analyzed when possible. When at least two studies reported the same factor with its corresponding odds ratio from a multivariable regression analysis, we combined such effect estimates (adjusted odds ratios) to produce a pooled OR in a meta-analysis. To meta-analyze these precalculated effect estimates, we used the log-odds ratios with their 95% confidence intervals as inputs to the analysis; however, the results of these meta-analyses are presented on the ratio scale (adjusted ORs with 95% CI).

In both meta-analyses (proportions and pre-calculated effect sizes), heterogeneity was evaluated using the I^2^ test, corresponding to low (I^2^ < 25%), medium (I^2^ = 25–75%), and high (I^2^ > 75%) heterogeneity.

We did not assess for publication bias because traditional methods (the funnel plot) have been proven inaccurate and unreliable for meta-analysis of proportions, such as in this case [16].

### 2.9. Subgroup Analyses and Meta-Regression

To explore the heterogeneity of the pooled prevalence obtained from the proportion meta-analysis, we performed subgroup analyses stratified by study design, geographical location, and NAFLD and PCOS diagnostic methods/criteria.

Using the effect estimates (estimated prevalence) and its standard errors obtained from the proportion meta-analysis, we performed a meta-regression analysis to explore if selected study-level summary data could influence the estimated prevalence.

We extracted the prevalence of metabolic syndrome and the mean values of HOMA-IR, free androgen index, and total testosterone (nmol/L) from the studies where they were available. We assumed that any effect on the estimated prevalence was mediated through changes in the variables of interest. Therefore, we performed a random-effects meta-regression to estimate the coefficients, β, which indicate how the estimated prevalence changed with a unit increase in the proposed explanatory variables. A REML algorithm estimated the between-study variance in this model. The results of the meta-regression were presented in “Bubble plots” with fitted meta-regression lines, with circles representing the estimates from each study, sized according to each estimate’s precision.

All statistical analyses were performed in Stata statistical software v.14.

## 3. Results

**Figure 1:**
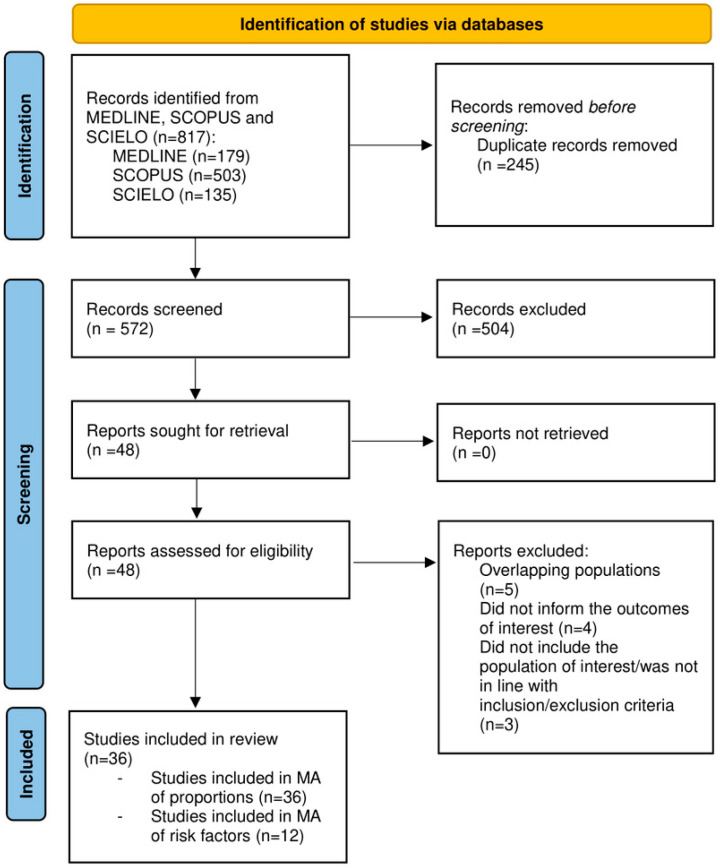
PRISMA diagram flow chart.

We identified 817 articles from the electronic database searches, of which 48 were considered eligible for inclusion in the present SR. After applying all inclusion and exclusion criteria, 36 were included [19,20,21,22,23,24,25,26,27,28,29,30,31,32,33,34,35,36,37,38,39,40,41,42,43,44,45,46,47,48,49,50,51,52,53,54]. All articles were included in the meta-analysis of the pooled NAFLD prevalence. Of the 36 studies, thirteen investigated the risk factors for NAFLD among PCOS patients; 12 were included in the meta-analysis of risk factors [27,28,29,30,33,34,37,38,39,42,44,47]. Figure 1 shows the PRISMA diagram for the selection of the studies.

### 3.1. Characteristics of the Included Studies

As shown in Table 1, the 36 articles included in this SR were published between 2007 and 2022. Of these, seventeen recruited participants from Asia, eight from Europe, six from Latin America, and five from the United States and Canada. Fourteen (39%) and thirteen (36%) papers presented case–control and cross-sectional study designs, respectively. In addition, six studies were case series (17%), and three (8%) were cohorts.

Tables S1 and S2 in the Supplementary File show that the studies were homogeneous regarding the populations analyzed, as they had similar inclusion and exclusion criteria and used comparable and appropriate PCOS and NAFLD diagnostic strategies. In addition, they all included cohorts of PCOS patients without known hepatitis B or C infection nor significant/harmful alcohol use. Therefore, we considered the studies included combinable and relevant for the qualitative and quantitative synthesis required for the present SR and MA.

### 3.2. Characteristics of Participants

As mentioned earlier, we extracted and charted data only from PCOS patients. The studies included in this SR recruited 7374 individuals, of which 5021 had a diagnosis of PCOS and 2156 were controls without PCOS. Table 1 presents the reported values of age, comorbidities, and relevant clinical and anthropometric data for PCOS patients.

The diagnosis of PCOS was similar across studies. In 31 (86%), the Rotterdam criteria were used to diagnose PCOS. The NIH criteria were used in 4 (11%) studies. One study reported using the Androgen Excess and PCOS Society criteria to diagnose the condition.

The burden of comorbidities was relevant in the studies that presented data on it. For example, in 13 studies that reported metabolic syndrome data, the prevalence of this condition ranged from 29% to 50%. Similarly, obesity prevalence was higher than 50% in half of the studies reporting data about it (*n* = 15) (Table 1).

### 3.3. NAFLD Assessment

As shown in Table 1, the diagnostic methods used to detect NAFLD were similar across studies. The most used diagnostic modality was ultrasound in 26 studies (72%). Transient elastography (TE), CT-scan, and MRI were used in four, two, and one study, respectively. Two studies reported using non-invasive blood biomarkers and panels (Hepatic steatosis index in one and NAFLD liver fat score in one). One study reported the use of several NAFLD diagnostic methods (ultrasound/fTE/MRI). A more detailed description of the definitions and cut-off values used to diagnose NAFLD is available in Table S2 in the Supplementary File.

Overall, of the PCOS patients included for analysis (*n* = 5021), 2072 (41.2%) were diagnosed with NAFLD.

#### Quantitative Synthesis (Meta-Analysis)

***Primary outcome: NAFLD prevalence***

**Figure 2:**
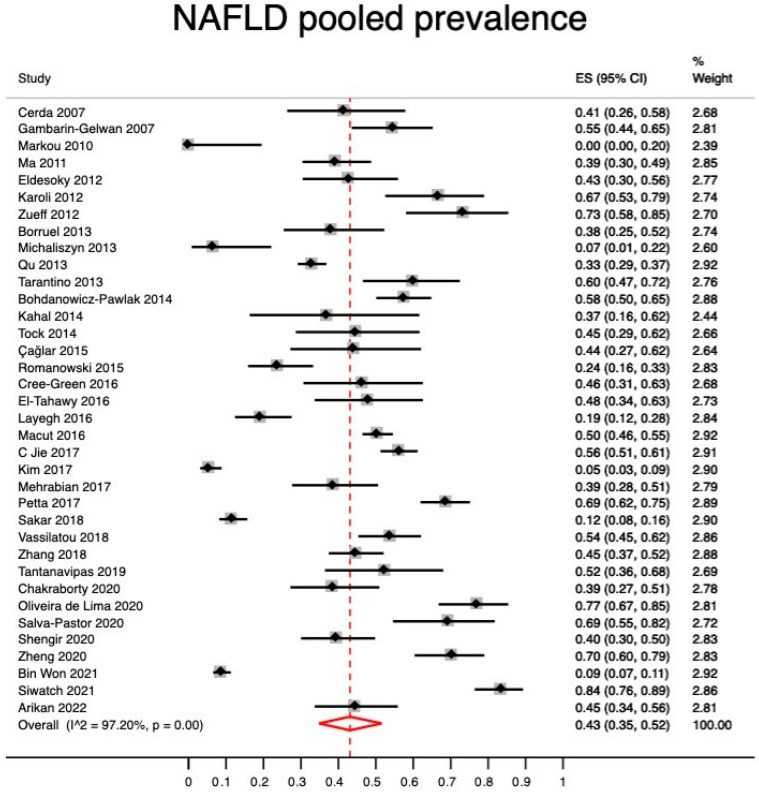
Pooled prevalence estimation of NAFLD in patietns with PCOS. Ref: [19,20,21,22,23,24,25,26,27,28,29,30,31,32,33,34,35,36,37,38,39,40,41,42,43,44,45,46,47,48,49,50,51,52,53,54].

Using a meta-analysis of proportions with a random-effects model, we found a pooled NAFLD prevalence of 43% (95% CI, 35–52) with high heterogeneity (I^2^ = 97.2%) (Figure 2).

***Sub-group analyses***

We performed subgroup analyses to explore the pooled NAFLD prevalence by a number of categorical study characteristics. Table 2 shows the results of subgroup analyses which were performed by study design, the region where the study was conducted, and the NAFLD and PCOS diagnostic methods/criteria used.

***Meta-regression analysis***

**Figure 3:**
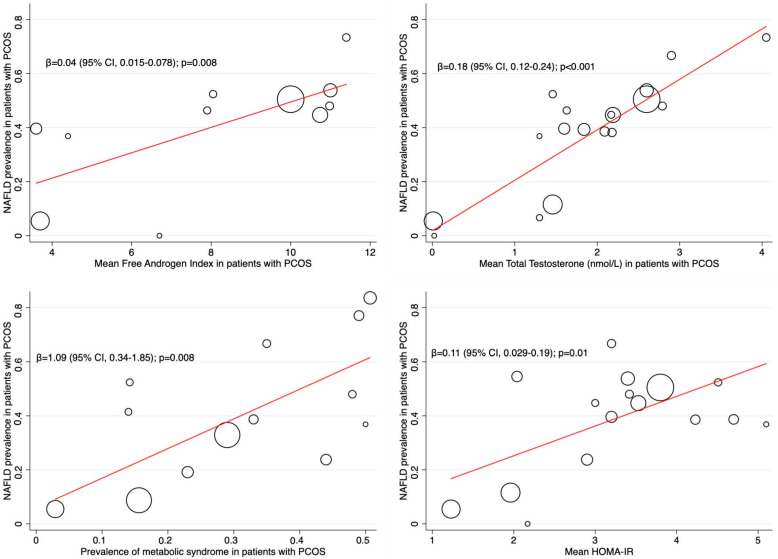
Metaregression analysis.

Meta-regression analyses showed that higher values of free androgen index (β = 0.04; 95% CI: 0.015–0.078; *p* = 0.008), total testosterone (β = 0.18; 95% CI: 0.12–0.24; *p* < 0.001), and HOMA-IR (β = 0.11; 95% CI: 0.029–0.19; *p* = 0.01) influenced increases in the prevalence of NAFLD among PCOS patients. Similarly, a higher prevalence of metabolic syndrome was associated with a higher prevalence of NAFLD (β = 1.09; 95% CI: 0.34–1.85; *p* = 0.008) (Figure 3).

***Meta-analysis of risk factors for NAFLD in patients with PCOS***

Of the 36 studies included in the SR, 12 reported effect estimates resulting from multivariable regression analyses and appropriate to combine in a meta-analysis. The factors pooled in the meta-analyses of risk factors were BMI, waist circumference, ALT values, HOMA-IR values, HDL levels, free androgen index levels, hyperandrogenism, and triglycerides. As shown in Table 2, all these factors, except for high-density lipoprotein levels, were associated with significantly higher risk-adjusted odds of NAFLD among patients with PCOS.

## 4. Discussion

After synthesizing data from 36 studies published worldwide (*n* = 5021 PCOS patients), two key points can be extracted: (1) the burden of NAFLD in PCOS patients is concerning, with an estimated pooled prevalence of 43% (95% CI, 35–52%); (2) obesity (BMI and waist circumference), metabolic abnormalities (HOMA-IR, ALT, and triglycerides), and PCOS specific hallmarks (hyperandrogenism and free androgen index) were identified as risk factors for NAFLD in PCOS populations. Meta-regression analysis showed metabolic features and PCOS-specific characteristics as potential effect modifiers, with rises in NAFLD prevalence mediated through increases in metabolic syndrome prevalence and higher levels of HOMA-IR, free androgen index, and total testosterone (Figure 3); thus, supporting the findings from the MA on risk factors.

This MA found a 43% NAFLD prevalence in patients with PCOS. This is concerning since it is higher than NAFLD prevalence previously reported in the general population and women globally. Younossi et al. [1] synthesized data from more than 8 million patients and found a pooled overall global prevalence of NAFLD of 25.2%. Although sex-specific data from a study showed an increase in the prevalence of NAFLD among females from 6.4% in 1990 to 8.4% in 2017 [55], it was still lower than our reported prevalence in those with PCOS. This suggest that females with PCOS have a higher burden of NAFLD than those without PCOS. Indeed, previous research found that PCOS is a risk factor for NAFLD [56,57]. Therefore, the NAFLD pooled prevalence in PCOS reported herein should inform stakeholders about the high burden of metabolic fatty liver disease in PCOS so they can design and deliver services to enhance prevention and implement nutritional and behavioral interventions with the final aim of reducing liver fat content. Thus, reducing the risk of disease progression towards non-alcoholic steatohepatitis and cirrhosis. In addition, NAFLD screening programs designed explicitly for PCOS patients could become a cornerstone for the early identification of metabolic liver disease.

It has been reported that the prevalence of NAFLD increases with age, affecting more than 40% of individuals older than 60 years [58,59]. Usually, women are not affected by it while they are pre-menopausal; however, as women reach the age of 50, NAFLD becomes more prevalent, with a peak near the age of 70. In contrast to what is already known, this review estimated a 40% prevalence of NAFLD in female populations with reported mean ages under 35 years. It is unlikely that young, healthy women present NAFLD features, and it is even less probable that they progress to advanced NAFLD stages, including NASH with significant fibrosis. However, in the presence of PCOS hyperandrogenemia, the liver is more likely to be infiltrated by fat, and NAFLD progresses faster. The NAFLD burden in PCOS is significant in at least two major respects. First, if left untreated, PCOS patients with established NAFLD may progress more rapidly to more advanced NAFLD stages, including NASH and cirrhosis [60]. Second, not only the incidence of NASH and cirrhosis could be increased in patients with PCOS, but the risk of mortality and poor hepatic and non-hepatic outcomes could be higher at ages where it usually is not, with the added peril that NASH is histologically more severe in females when compared to males [58,59]. Further work is required to establish the burden of liver-related outcomes in the PCOS population. The studies synthesized in this review did not report clinically relevant outcomes related to more advanced NAFLD stages.

The results of the risk factors MA indicate that the values of BMI, waist circumference, ALT, HOMA-IR, free androgen index, and triglycerides, and the presence of hyperandrogenism were all associated with significantly higher risk-adjusted odds of NAFLD among patients with PCOS. We acknowledge that, albeit statistically significant, the estimated odds ratios and 95% CIs for several of these factors were very low, meaning that the associations described were weak, as shown by the trivial effect size. In contrast, meaningful effect sizes were found for BMI, HOMA-IR values, and hyperandrogenism, which have been shown to interact in complex pathophysiological pathways leading to defective metabolic states with the inherent risk of unsatisfactory metabolic and cardiovascular outcomes. For example, elevated levels of androgens in PCOS patients disrupt normal physiology [6,61,62], causing dyslipidemia, hyperinsulinemia, insulin resistance, and increased visceral fat accumulation with adipose tissue dysfunction [63]. At the same time, dyslipidemia, and adipose tissue abnormalities also contribute to hyperinsulinemia and insulin resistance [64,65], risk factors for a cluster of related metabolic diseases, known as metabolic syndrome, with the liver as one of its metabolically affected organs. Therefore, it is rational to think of obesity (BMI), insulin resistance (HOMA-IR), and hyperandrogenism as mechanistically plausible causes of NAFLD in PCOS patients. Furthermore, meta-regression results are consistent with these assumptions revealing that increases in metabolic syndrome prevalence, HOMA-IR and free androgen index, and total testosterone values mediated increases in NAFLD prevalence, thus further supporting the associations found in the risk-factors MA.

The relevance of elevated androgen levels in the development of NAFLD in PCOS patients has been previously described [4,5,6] and is supported by the findings from the risk factors MA and meta-regression, which revealed that hyperandrogenism and free androgen index were associated with higher-risk adjusted odds of NAFLD, and that increased levels of FAI and total testosterone mediated higher NAFLD prevalence rates in PCOS populations. Perhaps hyperandrogenism, the hallmark feature of PCOS, is the most significant driver of the relationship between PCOS and NAFLD, and thus, specific NAFLD-in-PCOS prediction scores, including hyperandrogenism, should be developed to forecast NAFLD among patients affected by PCOS. Meanwhile, caregivers could use the NAFLD risk factors presented herein as elements that, if present, should underpin their awareness of the risk of NAFLD to offer efficient pathways to screen and detect it by using the resources available in their healthcare system. Moreover, PCOS stakeholders should start designing, implementing, and testing NAFLD screening programs focused on patients affected by PCOS.

### Limitations

This meta-analytic study has limitations and results should be interpreted in the context of the study design. First, one source of weakness in this study that could have affected the validity of the NAFLD prevalence estimates presented was the high heterogeneity found in the meta-analyses of proportions, maintained even in subgroup analyses. These heterogeneity levels may limit the generalizability of the calculated pooled prevalence. However, we synthesized data from “combinable” studies as inclusion and exclusion criteria and diagnostic NAFLD and PCOS methods were similar across studies, meaning the amount of clinical heterogeneity was low. In this scenario, methodological studies [66] have demonstrated that a quantitative synthesis is appropriate, even with extreme heterogeneity, such as in this MA. Moreover, combining data from 36 studies provides a more precise and reliable prevalence estimate than any individual proportion contributing to the meta-analytic pooled analysis.

Second, although inclusion and exclusion criteria and diagnostic strategies were similar across studies, the data synthesized came from a subset of studies with heterogeneous methodological quality, which inevitably permeated the SR and its results with bias arising from primary studies, making the meta-analysis at higher risk of meta-bias [67]. Additionally, we should have attempted to explore the grey literature to reduce the chance of further meta-bias.

Finally, it is still not known if PCOS patients have a higher risk of NASH/cirrhosis-related outcomes. What is less clear is if these risks may appear at younger ages. In other words, it is unknown if PCOS females may eventually require more advanced medical care, emergency department visits, hospitalizations, and even liver transplantation earlier. Therefore, more work is required to come upon the natural history of NAFLD in PCOS.

Despite its limitations, this systematic review certainly adds to our understanding of the burden and the factors associated with NAFLD in PCOS. However, more work will need to be done to determine how the factors described in the meta-analytic analyses could cause specific biological effects. Furthermore, the issue of prediction scores and screening programs designed to forecast and detect NAFLD in PCOS is paramount, and could be usefully explored in further research.

While NAFLD-in-PCOS research advances, patients should be offered care bundles, including nutritional counseling, physical exercise, and oral contraceptives. In the case of detecting NAFLD, the stage of liver fibrosis should be evaluated and, if necessary, a liver biopsy performed. In the event of NASH, a hepatologist should set the discussion about the feasibility of inclusion in a NAFLD/NASH randomized clinical trial.

## 5. Conclusions

The results presented in this SR and MA portray a worrying scenario with a high prevalence of NAFLD (43%) among PCOS patients with several metabolic and PCOS-specific factors influencing its occurrence. As the burden of NAFLD appears to be high, screening programs may aid in detecting metabolic-associated fatty liver disease, and prevent its consequences in a population where this condition has been commonly overlooked.

## Figures and Tables

**Table 1:** Study and Patients’ characteristics.

Author/y	Study Design	Country	PCOS Dx	NAFLD Dx	N Total	N PCOS	N NAFLD in PCOS	Age	BMI kg/m^2^	TG mg/dL	Metabolic Syndrome, *n* (%)	Obesity, *n* (%)	T2DM, *n* (%)	Weight Circumference (cm)	HOMA-IR	FAI	Total Testosterone nmol/L
[51] Cerda 2007	Cohort	Chile	Rotterdam	US	72	41	17	24.6 (7.2)	30.3 (7.07)	125.5 (96.6)	6 (14.6)	24 (58.5)	3 (7.3)	NR	NR	NR	NR
[54] Gambarin-Gelwan 2007	Case-series	USA	NIH	US	88	88	48	31.4	26.9	97	NR	NR	NR	NR	2.04	NR	NR
[26] Markou 2010	Case-control	Greece	Rotterdam	CT-Scan	34	17	0	25.1 (1)	20.9 (0.5)	55.7 (5.2)	NR	NR	NR	73.1 (2.3)	2.17 (0.29)	6.7 (1.1)	0.023 (0.0027)
[29] Ma 2011	Cross-sectional	China	Rotterdam	US	117	117	46	NR	NR	NR	NR	NR	NR	NR	NR	NR	1.84 (0.9)
[19] Eldesoky 2012	Case-series	Egypt	Rotterdam	US	63	63	27	NR	NR	NR	NR	46 (73)	18 (28.5)	NR	NR	NR	NR
[22] Karoli 2012	Cross-sectional	India	Rotterdam	US	109	54	36	28.5 (6.2)	27.2 (5.4)	136 (24)	19 (35)	NR	NR	NR	3.2 (1.7)	NR	2.9 (0.7)
[48] Zueff 2012	Case-control	Brasil	Rotterdam	US	90	45	33	31.6 (4.1)	34.7 (2.9)	102.5 (80.5–163)	NR	NR	NR	103.71 (8.83)	NR	10.4 (6.9–16.9)	2.97 (2.13–7.06)
[49] Borruel 2013	Case-control	Spain	NIH	US	106	55	21	NR	NR	83 (39)	NR	23 (42)	NR	88 (20)	NR	NR	2.18 (0.86)
[32] Michaliszyn 2013	Case-series	USA	NIH	CT-Scan	30	30	2	16.1 (03)	37.1 (1.3)	NR	NR	NR	NR	104.5 (3.3)	NR	NR	1.3 (0.1)
[35] Qu 2013	Case-series	China	Rotterdam	US	602	602	198	NR	NR	NR	NR	NR	NR	NR	NR	NR	NR
[43] Tarantino 2013	Cross-sectional	Italy	Rotterdam	US	80	60	36	25.5 (16–38)	25.2 (18.2–46.6)	158.5 (52–230)	NR	30 (50)	NR	85 (67–118)	2.6 (0.7–11.1)	9.1 (3.4–16.9) no sirve	3.8 (1.8–5.48)
[42] Bohdanowicz-Pawlak 2014	Cross-sectional	Poland	Rotterdam	US	184	184	106	NR	NR	NR	NR	NR	NR	NR	NR	NR	NR
[21] Kahal 2014	Case-control	UK	Rotterdam	US	36	19	7	33.9 (6.7)	37.9 (5)	NR	10 (50)	19 (100)	NR	112 (12.6)	5.1 (2.6)	4.4 (2.2)	1.3 (0.4)
[44] Tock 2014	Case-series	Brasil	Rotterdam	US	38	38	17	28.3 (6.8)	32.9 (7.7)	108 (48.4)	NR	NR	NR	103.2 (19.2)	3 (2.3)	NR	2.17 (1.25)
[50] Çağlar 2015	Case-control	Turkey	Rotterdam	US	55	34	15	26 (2.5)	22 (1.1)	80 (34–233)	NR	NR	NR	NR	2 (0.8–14)	NR	0.01 (0.001–0.045)
[36] Romanowski 2015	Case-control	Brasil	AE and PCOS society	US	131	101	24	26.8 (5)	28.5 (6)	103.3 (60)	45 (44.6)	NR	NR	91.6 (16)	2.9 (2)	NR	NR
[52] Cree-Green 2016	Cohort	USA	NIH	MRI	71	41	19	15 (13–16)	35.2 (0.61)	122 (76–158)	NR	19 (100)	NR	103 (96–111)	NR	7.9 (6.6–14.6) si sirve	1.63 (1.17–2.11)
[53] El-Tahawy 2016	Case-control	Egypt	Rotterdam	US	105	50	24	28.3 (5.4)	29.8 (6.7)	130.3 (18.9)	24 (48)	NR	2 (4)	NR	3.42 (0.87)	10.98 (5.73)	2.79 (0.87)
[24] Layegh 2016	Cross-sectional	Iran	Rotterdam	US	115	115	22	24.5 (5.4)	NR	NR	27 (23.4)	70 (60.8)	NR	NR	NR	NR	NR
[25] Macut 2016	Cross-sectional	Serbia	Rotterdam	NAFLD fatty liver score	725	600	303	25.6 (25.1–26.1)	30.7 (30.1–31.3)	NR	NR	Nr	NR	91.8 (90.6–92.9)	3.8 (3.6–4)	10 (9.4–10.6)	2.6 (2.5–2.7)
[28] C Jie 2017	Cross-sectional	China	Rotterdam	Ultrasound	500	400	225	25.8	NR	NR	NR	NR	NR	NR	NR	NR	NR
[23] Kim 2017	Case-control	Korea	Rotterdam	Ultrasound	1167	275	15	30.4 (5.2)	20.3 (2.1)	66 (51–86)	8 (2.9)	0	0	75 (6)	1.23 (1.09–1.37)	3.7 (2.5–4.9) si sirve	0.011 (0.008–0.014)
[30] Mehrabian 2017	Cross-sectional	Iran	Rotterdam	US	150	75	29	NR	24.7 (1.7)	149.7 (37.4)	25 (33.3)	NR	NR	86.5 (8.8)	4.7 (1.8)	NR	NR
[34] Petta 2017	Case-control	Italy	Rotterdam	Hepatic steatosis index	303	202	139	33.2 (5.5)	25.7 (2.9)	112.6 (44)	NR	97 (48.2)	NR	87.5 (22)	NR	NR	NR
[38] Sarkar 2018	Cohort	USA	Rotterdam	TE	303	303	35	28.2 (6.8)	27.6 (10.7)	81 (69)	NR	NR	NR	83.82 (25.4)	1.96 (2.4)	NR	1.46 (1.79)
[45] Vassilatou 2018	Case-control	Greece	Rotterdam	US	290	145	78	27.5 (7.1)	31.8 (6.9)	NR	NR	NR	NR	93.7 (15.4)	3.4 (2.1)	11 (6.7)	2.6 (0.9)
[46] Zhang 2018	Case-control	China	Rotterdam	US	253	188	84	27.1 (5.2)	25.1 (3.2)	NR	NR	74 (39.3)	NR	NR	3.53 (0.64)	10.82 (8.89–12.51) si sirve	2.19 (0.88)
[41] Tantanavipas 2019	Case-control	Thailand	Rotterdam	US	63	42	22	27.7 (5.2)	27.05 (6.5)	104.3 (71.9)	6 (14.2)	NR	NR	84.81 (14.7)	4.51 (4.97)	8.05 (8.17)	1.46 (0.74)
[27] Chakraborty 2020	Cross-sectional	India	Rotterdam	US	130	70	27	20.4 (2.4)	25.1 (4.6)	98.9 (37.6)	NR	NR	NR	NR	4.23 (5.1)	NR	2.09 (0.81)
[33] Oliveira de Lima 2020	Case-series	Brasil	Rotterdam	US	127	87	67	34.4 (5.7)	34.7 (4.7)	134 (49–373)	43 (49.9)	75 (86.2)	11 (12.6)	103 (67–128) no sirve	NR	NR	NR
[37] Salva-Pastor 2020	Case-control	Mexico	Rotterdam	TE	98	49	34	NR	NR	NR	NR	NR	NR	NR	NR	NR	NR
[39] Shengir 2020	Cross-sectional	Canada	Rotterdam	TE	101	101	40	36.3	27.6 (5)	NR		97 (96)	18 (17.8)	101.1 (12.3)	3.2 (2.9)	3.6 (3.7)	1.6 (0.7)
[47] Zheng 2020	Cross-sectional	China	Rotterdam	TE	101	101	71	NR	NR	NR	NR	77 (76.2)	NR	NR	NR	NR	NR
[31] Bin Won 2021	Cross-sectional	Korea	Rotterdam	US/TE/MRI	586	586	51	NR	23.8	NR	92 (15.6)	198 (33.7)	NR	NR	NR	NR	NR
[40] Siwatch 2021	Cross-sectional	India	Rotterdam	US	210	140	117	27.4 (3.5)	25.6 (4.1)	NR	71 (50.7)	76 (54.3)	NR	NR	NR	NR	NR
[20] Arikan 2022	Case-control	Turkey	Rotterdam	US	141	83	37	24.7 (6.2)	24.5 (4.7)	107 (69.2)	NR	NR	NR	83.6 (13.1)	NR	NR	NR

MRI: magnetic resonance imaging; NR: not reported; TE: transient elastography; US: ultrasound; TG: triglycerides; FAI: free androgen index; Data are presented in mean (SD) or median (IQR) as reported in the studies.

**Table 2:** Results from subgroups analyses (meta-analysis of proportions) and risk factors meta-analysis.

*Part 1. Subgroup Analyses (Meta-Analysis of Proportions)*		
Subgroups	Pooled Prevalence (95% CI)	Heterogeneity (I^2^)
**Study design**		
Case-Control (14 studies)	41% (27–57)	96.50%
Cross-Sectional (13 studies)	48% (33–63)	98.14%
Case-Series (6 studies)	43% (20–60)	94.54%
Cohort (3 studies)	31% (8–60)	Not estimable
**Region**		
Asia (15 studies)	42% (28–56)	98.15%
Europe (8 studies)	48% (38–58)	89.36%
Latin America (6 studies)	55% (35–75)	93.41%
USA and Canada (5 studies)	30% (11–52)	95.81%
Africa (2 studies)	45% (36–54)	Not estimable
**NAFLD assessment method**		
Ultrasound (26 studies)	46% (38–55)	95.61%
Transient elastography ( 4studies)	46% (15–79)	98.23%
CT-Scan (2 studies)	3% (0–11)	Not estimable
Non-invasive blood biomarkers/Panels (2 studies)	55% (52–59)	Not estimable
MRI (1 study)	46% (31–53)	Not estimable
**PCOS diagnostic criteria**		
Rotterdam (31 studies)	45% (36–54)	97.52%
NIH (4 studies)	35% (17–57)	89.09%
AE and PCOS society (1 study)	24% (16–33)	Not estimable
***Part 2. Risk Factors Meta-analysis***		
**Factor**	**Odds Ratio (95% CI)**	**Heterogeneity, I^2^**
BMI (8 studies)	1.35 (1.28–1.430)	70.00%
Waist circumference (5 studies)	1.016 (1.006–1.027)	71.60%
ALT (4 studies)	1.007 (1.001–1.014)	81.40%
HOMA -IR (4 studies)	1.21 (1.09–1.24)	36.50%
HDL (2 studies)	0.99 (0.96–1.03)	72.10%
Free Androgen Index (2 studies)	1.06 (1.03–1.1)	82.30%
Hyperandrogenism (2 studies)	10.3 (4.2–25.2)	58.10%
Triglycerides (2 studies)	1.002 (1.001–1.004)	63.60%

## Data Availability

The data and Stata commands used to perform the present systematic review, meta-analysis, and meta-regression can be shared upon reasonable request.

## Supplementary Materials

The following supporting information can be downloaded at: https://www.mdpi.com/article/10.3390/jcm12030856/s1, 1. Search Strategies, 2. Risk of Bias within studies: Figure S1. Risk of bias evaluation according to MINORS, 3. Table S1. Objectives, inclusion, and exclusion criteria from each study, 4. Table S2. Definitions and cut-off values used to diagnose NAFLD, 5. Meta-analysis of Observational Studies in Epidemiology (MOOSE) Checklist.
