# Supplementary material for: Non-Alcoholic Fatty Liver Disease in Patients with Polycystic Ovary Syndrome: A Systematic Review, Meta-Analysis, and Meta-Regression
Source: J Clin Med. 2023 Jan 20;12(3):856. doi: 10.3390/jcm12030856 (PMC9917911; doi:10.3390/jcm12030856)
Supplement: Supplementary file 1 [file jcm-12-00856-s001.zip › jcm-2120360-supplementary.pdf]

Supplementary File

The present electronic supplementary material was prepared for the manuscript **"Non-alcoholic fatty liver disease in patients with polycystic ovary syndrome: a systematic review, meta-analysis, and meta-regression"**. For questions about the information herein contained, please get in touch with the corresponding author:

Juan M. Pericas
e-mail address: juanmanuel.pericas@vallhebron.cat, juan.pericas@vhir.org
Liver Unit
Internal Medicine Department
Vall d'Hebron University Hospital
Barcelona, Spain

This supplement contains the following items:

1. Search Strategies

2. Risk of Bias within studies: Figure S1. Risk of bias evaluation according to MINORS

3. Table S1. Objectives, inclusion and exclusion criteria from each study

4. Table S2. Definitions and cut-off values used to diagnose NAFLD

5. Meta-analysis of Observational Studies in Epidemiology (MOOSE) Checklist

1. Search Strategies:

Searches were performed on 6/10/2022

MEDLINE:

1. exp Polycystic Ovary Syndrome/
2. PCOS.mp.
3. polycystic ovarian disease.mp.
4. Stein-Leventhal syndrome.mp.
5. exp Fatty Liver/
6. exp Non-alcoholic Fatty Liver Disease/
7. NASH.mp.
8. nonalcoholic steatohepatitis.mp.

9. liver ultrasound.mp.
10. hepatic ultrasound.mp.
11. hepatic ultrasonography.mp.
12. liver ultrasonography.mp.
13. fibroscan.mp.
14. Vibration-controlled transient elastography.mp.
15. Controlled attenuation parameter.mp.
16. 1 or 2 or 3 or 4
17. 5 or 6 or 7 or 8 or 9 or 10 or 11 or 12 or 13 or 14 or 15
18. 16 and 17

SCOPUS: (TITLE-ABS-KEY ("Polycystic ovary syndrome") OR TITLE-ABS-KEY (pcos) OR TITLE-ABS-KEY ("polycystic ovarian disease") AND TITLE-ABS-KEY ("non-alcoholic fatty liver disease") OR TITLE-ABS-KEY (nafld) OR TITLE-ABS-KEY (nash) OR TITLE-ABS-KEY ("nonalcoholic steatohepatitis") OR TITLE-ABS-KEY ("liver ultrasound") OR TITLE-ABS-KEY ("hepatic ultrasound") OR TITLE-ABS-KEY (fibroscan) OR TITLE-ABS-KEY ("controlled attenuation parameter") OR TITLE-ABS-KEY ("Vibration-controlled transient elastography") OR TITLE-ABS-KEY ("fatty liver"))

SCIELO: ("polycystic ovary syndrome") OR (PCOS) AND ("Fatty liver") OR (NAFLD) OR (NASH) OR ("nonalcoholic fatty liver disease") OR ("nonalcoholic steatohepatitis")

2. Figure S1. Risk of bias evaluation according to MINORS. Ref: [19-54]

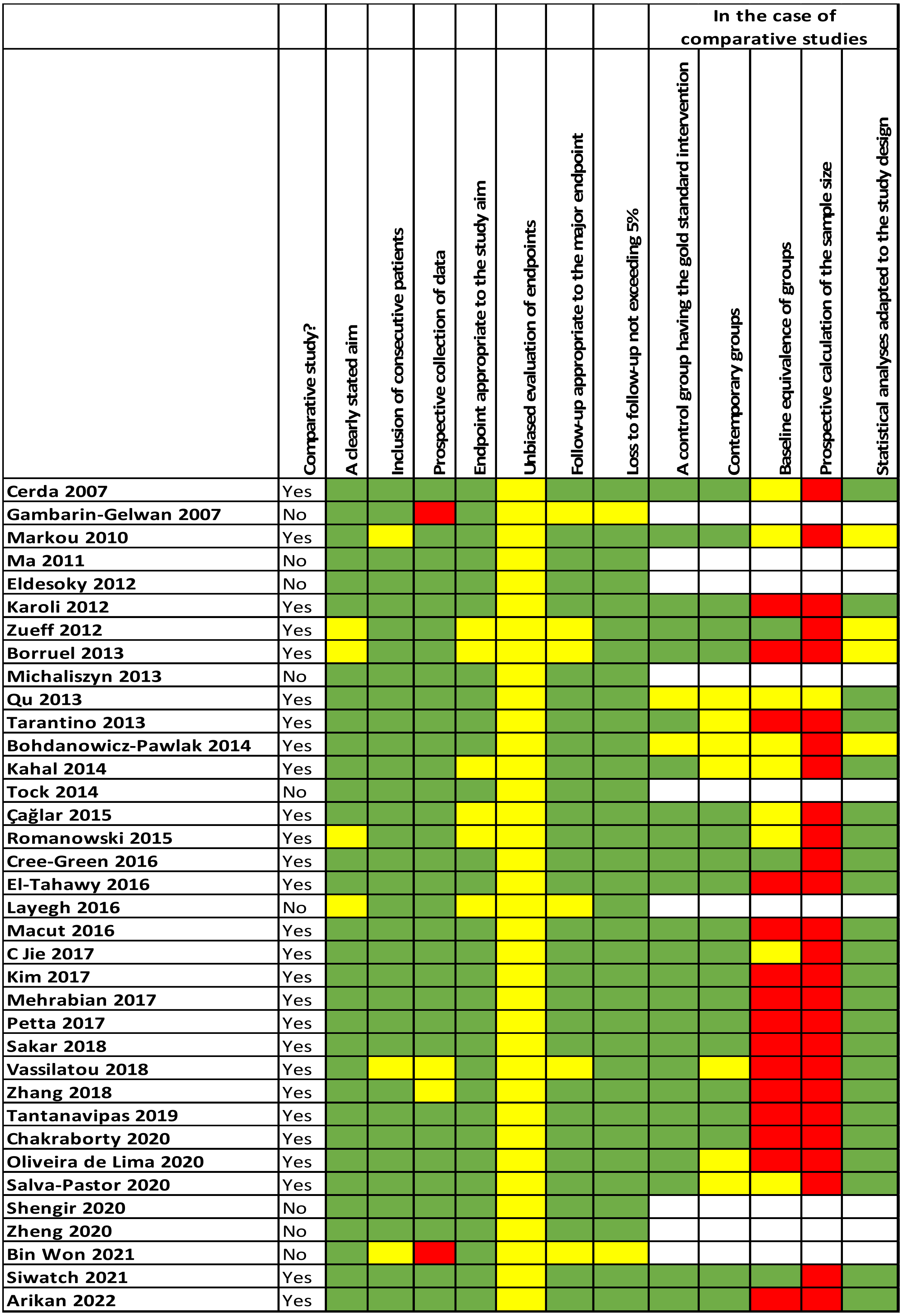

3. Table S1. Objectives, inclusion and exclusion criteria from each study

Author/y	Objective	Population/Inclusion criteria	Exclusion criteria	Included pts with viral hep	Included pts with significant alcohol consumption
[51] Cerda 2007	The current prospective study was conducted with the aim of determining the frequency and characteristics of NAFLD in Chilean women with PCOS	Non-pregnant patients with diagnosis of PCOS and no current pharmacological treatment, attending an endocrinology clinic	Subjects were excluded if they had history of alcohol intake higher than 20g per day. Women with a history of chronic viral hepatitis, hemochromatosis, autoimmune liver disease, other chronic liver disease, or those taking hepatotoxic drugs were excluded.	No	No
[54] Gambarin-Gelwan 2007	The aim of this study was to estimate the prevalence of NAFLD in women with PCOS by using abdominal ultrasonography and to note the presence and severity of hepatic steatosis in both obese and nonobese women with PCOS. An additional goal was to identify factors associated with NAFLD in women with PCOS.	Women were included in the study population if they were 18 years of age or older at the time of the initial evaluation and met the consensus diagnostic criteria for PCOS from the 1990 National Institutes of Health conference in Bethesda, Maryland: (1) menstrual dysfunction, (2) hyperandrogenism, and (3) the exclusion of other causes.	Subjects were excluded if they had a history of heavy alcohol use, defined as greater than 2 drinks or 20 g alcohol per day. Women with a history of chronic viral hepatitis, hemochromatosis, other chronic liver disease, or those taking hepatotoxic drugs were excluded.	No	No
[26] Markou 2010	In the present study we investigated the presence of NAFLD in young lean women with PCOS and IR, using both US and CT scans as radiologic means of assessing hepatic lipid content to obtain maximum sensitivity and diagnostic accuracy.	Women who were attending the outpatient endocrinology clinic and had a diagnosis of PCOS, according to the Rotterdam criteria	Exclusion criteria were the following: 1) other causes of irregular menstrual cycles and androgen excess or pregnancy, 2) history of known liver disease or other medical condition or any medication known to cause an elevation in liver enzymes, 3) alcohol consumption of more than 14 units per week (>20 g daily)	No	No
[29] Ma 2011	The aim of our study is to evaluate the relationship between visceral adiposity, as measured by noninvasive sonographic techniques, and metabolic parameters and fatty liver among	Subjects with PCOS	Exclusion criteria included the use of oral contraceptives or corticosteroids within the past 3 months. All subjects had alcohol intake of less than 20 g of ethanol per day, as assessed by a structured questionnaire concerning dietary habits and medical history conducted by a research	No	No

	Chinese women with PCOS.		nurse. Subjects with hypothyroidism, prolactinoma, nonclassical adrenal hyperplasia, and Cushing's syndrome were excluded from the study. In addition, all subjects with hepatitis B were excluded from the study.		
**[19] Eldesoky 2012**	We aimed to study the prevalence and characteristics of NAFLD in young adult Egyptian women with PCOS	Adult nontreated women with PCOS, ranging in age from 23 to 37 years/Patients were included in this study if they had history of irregular menstrual cycles and evidence of androgenic excess, including hirsutism, acne, male pattern hair loss, or an elevated testosterone level.	Pts were excluded if they had other causes of irregular menstrual cycles or androgenic excess including hyperprolactinemia, uncontrolled tyroid disease, congenital adrenal hyperplasia, premature ovarian failure, Cushing's syndrome, androgen-secreting tumor, or pregnancy. We also excluded pregnant women, smokers, and those with a history suggestive of chronic viral hepatitis, hemochrimatosis, wilson disease, other metabolic or autoimmune chronic liver disease,alcohol intake, or those taking ovulatory drugs or other known hepatotoxic agents	No	No
**[22] Karoli 2012**	We planned to undertake this study with the aim to identify which factors are associated with presence of NAFLD in this subset of patients. The patients with PCOS were evaluated with abdominal ultrasonography and biochemical testing in order to assess the presence of NAFLD and its association with various metabolic and hormonal factors was studied.	Premenopausal women with PCOS diagnosed by Rotterdam criteria. Women with any two of the following were included: oligomenorrhea/oligo-ovulation, clinical or biochemical hyperandrogenism and polycystic ovaries on ultrasound. Oligomenorrhea was defined as absence of menstruation ≥35 days, amenorrhea-no menstruation for >6 months.	Women excluded from the studywere those with inherited disorders of insulin resistance, type 2 diabetes mellitus, hypertension, Cushing's syndrome, hyperprolactinemia, untreated hypothyroidism, congenital adrenal hyperplasia, with an androgen-secreting adrenal/ ovarian tumor and those taking corticosteroids, antiepileptic or antipsychotic drugs, insulin sensitizers, hormonal contraceptives, antituberculosis drugs in past three months, currently pregnant or in the first postpartum year. The patients with alcohol consumption >20 gm/day, history of chronic viral hepatitis, hemochomatosis, autoimmune hepatitis, drug-toxin-induced liver injury or chronic liver disease were also excluded.	No	No

**[48] Zueff 2012**	In this study we evaluated and compared multiple ultrasound and laboratory markers of metabolic disorders and CVD between PCOS women and age-matched controls	Women aged between 18 and 40 with a BMI ≥30 and <40 kg/m2 seeking contraception were invited to participate in the study. We included all women with a diagnosis of PCOS, defined as the presence of two of the following criteria: oligo-ovulation and/or anovulation, clinical and/or biochemical signs of hyperandrogenism and polycystic ovaries (PCO) at ultrasound evaluation	The following were regarded as exclusion criteria: smoking; hypertension; history of deep venous thrombosis or pulmonary embolism; known thrombogenic mutations; current or history of ischemic heart disease; valvular heart disease; stroke; systemic lupus erythematosus; migraine; diabetes; gallbladder disease; viral hepatitis; hepatic cirrhosis; liver tumors; sickle cell disease; and use of anticonvulsant therapy. Since they could affect the outcome measures the following were also regarded as exclusion criteria: regular alcohol consumption (≥3 times/week); drug addiction; use of hormonal contraception (injectable within 6 months and other within 2 months) before the start of the study; and use of statins, antihypertensive drugs and antiretroviral drugs.	No	No
**[49] Borruel 2013**	We conducted a systematic ultrasound examination of the thickness of SAT, VAT, and organ-specific adipose tissue depots in patients with PCOS, control women showing no evidence of androgen excess, and healthy men.	We recruited prospectively 25 healthy control women without evidence of androgen excess, 55 patients with PCOS, and 26 healthy men selected as to have similar body mass index (BMI).	NR	NR	NR
**[32] Michaliszyn 2013**	We investigated in obese adolescent girls with PCOS the relationship between liver fat and in vivo insulin sensitivity, body composition, abdominal adiposity, and lipid metabolism.	Tanner stage V obese adolescents with PCOS	Exclusion criteria included pre-existing treatment for PCOS, pregnancy, existing systemic or psychiatric disease, and the use of medications that influence glucose or lipid metabolism or blood pressure.	NR	NR
**[35] Qu 2013**	The purpose of the current research is to study the prevalence and clinical features of NAFLD in Chinese	Non-pregnant women with PCOS	Women with a history of acute viral hepatitis, haemochromatosis, autoimmune liver disease, or other diseases were excluded.	No	No

	women with PCOS, to analysis risk factors for the prevalence of NAFLD in women with PCOS, the effect of BMI on hyperandrogenism on the prevalence of NAFLD in women with PCOS; and the screening index for NAFLD.				
**[43] Tarantino 2013**	The aim of our study was to evaluate the role of the spleen in determining LGCI and the weight of this organ in the relationship between PCOS and hepatis steatosis in a group of young women with PCOS according to body weight, IR, and PCOS phenotypes	Women with PCOS diagnosis. Patients were enrolled according to the following criteria: premenopausal status, age range (15-40) for diagnoses of PCOS; anovulatory oligo-amenorrhea; caucasian ethnicity	Patients were excluded according to the following criteria: smoking or alcohol consumption, pregnancy, hypothyroidism, hyperprolactinemia, Cushing's syndrome, non-classical congenital adrenal hyperplasia; previous (within the last 6 months) use of oral contraceptives, insulin sensitizing agents, glucocorticoids, anti-androgens, ovulation induction agents, anti-obesity drugs, presence of any acute viral, bacterial or fungal infection, chronic liver diseases of various nature, arthritis, bronchial asthma and chronic inflammatory bowel and cancer.	No	No
**[42] Bohdanowicz-Pawlak 2014**	The aim of this study was to determine whether there is an association between PCOS and NAFLD, and whether in women with PCOS LPL gene polymorphism is associated with the aetiology of metabolic disorders occurring in these women	Obese and overweight women affected by PCOS	Pregnancy, current or previous (within six months) use of oral contraceptives, anti-androgens or other hormonal drugs, known cardiovascular disease (CVD), diabetes mellitus, hypertension, cigarette smoking, history of liver diseases, and chronic alcohol consumption.	No	No
**[21] Kahal 2014**	The primary aim of this study was to investigate the effects of 6-month treatment with liraglutide on metabolic and markers of liver disease in obese women with PCOS and controls.	Obese women with PCOS and age- and weight-matched controls.	Other endocrine disorders with similar presentation were excluded. Control subjects with a history of clinical or biochemical hirsutism or menstrual irregularities were excluded. Other causes of liver disease were excluded by measuring serum alpha-1 antitrypsin, ceruloplasmin, ferritin, autoimmune profile and hepatitis B and C	No	No

			serology. Participants with alcohol intake of >14 units/week were excluded from the study.		
**[44] Tock 2014**	The primary objective of this study was to assess NAFLD in PCOS women with and without OSA. The secondary aim was to evaluate a possible role for hyperandrogenemia in the development of OSA in PCOS women.	PCOS subjects ranging 16 and 45 years. The diagnosis of PCOS was based on the 2003 Rotterdam criteria	Exclusion criteria included the use of oral contraceptives, corticosteroids, antidiabetic or lipid-lowering drugs in the past 3 months, history of liver disease such as viral hepatitis B and C, hemochromatosis and autoimmune hepatitis, use of medications that alter liver enzymes, and daily ingestion of more than 20 g of ethanol. We also excluded patients with diabetes mellitus, untreated hypothyroidism, renal, hepatic, cardiac or pulmonary disease, and patients being treated for sleep apnea and any other condition that could influence the polysomnography (PSG) test, such as drugs (sympathomimetics, sympatholytics, and β-blockers), depression, and chronic diseases.	No	No
**[50] Çağlar 2015**	We aimed to explore the relationship between insulin resistance (IR) and small dense lipoprotein (sd-LDL) particles, carotid intima-media thickness (CIMT) and non-alcoholic fatty liver disease (NAFLD) in young normal weight PCOS cases.	PCOS patients admitted an Out-patient Clinic to Obstetrics and Gynecology Department of University were enrolled in the study. The controls were healthy volunteers without any features of clinical or biochemical hyperandrogenism who had regular menstrual cycles.	Exclusion criteria were hyperprolactinemia, thyroid dysfunction, adrenal dysfunction, diabetes mellitus, pregnancy, alcohol consumption, history of chronic viral hepatitis, hemochromatosis, autoimmune liver disease, other chronic liver diseases, history of hepatotoxic, antihypertensive, lipid lowering or antiinflammatory agent usage	No	No
**[36] Romanowski 2015**	Prospective study developed to determine the prevalence of NAFLD and MS in adult women with PCOS.	Women aged ≥18 years old with confirmed diagnosis of PCOS based on criteria established by the Androgen Excess Society Guidelines, not using hormonal contraception for at least 3 months, were included	Patients who met at least one of the following criteria were excluded: a) previous history of chronic liver disease; b) suffering from conditions that can occur with known liver enzyme abnormalities; c) using medication that are risk factors for NAFLD such as corticosteroids, tamoxifen, amiodarone, diltiazem, protease inhibitors (ARVs); d) using metformin for hirsutism because it may interfere in blood glucose	No	No

			levels; e) daily consumption of ethanol ≥20 grams; f) failure to agree in participating in the study.		
**[52] Cree-Green 2016**	The aim of this study was to measure hepatic fat in nondiabetic youth with PCOS versus nondiabetic girls with regular menses who had equivalent obesity, as well as to examine mechanisms potentially contributing to HS.	Inclusion criteria were female sex, obesity (BMI percentile >95%), and sedentary status (<3 h of exercise/week; validated with both a 3-day activity recall and 7-day accelerometer use).	Exclusion criteria were diabetes, alanine transferase (ALT) >80 IU/mL, BP >140/ 90 mmHg, hemoglobin <9 mg/dL, serum creatinine >1.5 mg/dL, smoking, medications affecting IS (oral steroids, metformin, thiazolidinediones, atypical antipsychotics, hormonal contraceptives), antihypertensive medications, statins, pregnancy, and breastfeeding.	NR	NR
**[53] El-Tahawy 2016**	Our study aimed to identify which factors are associated with presence of NAFLD in PCOS' patients. Objectives: 1- Tto determine the rate of NAFLD in PCO. 2- To identify the risk factors of NAFL in PCO. 3-To determine histopathologial changes of NAFLD	Non-pregnant patients Diagnosed with PCOS and no current pharmacological treatment. Women aged ≥18 years old not using hormonal contraception for at least 3 months and with confirmed diagnosis of PCOS based on the revised criteria of ESHRE/ASRM	Congenital adrenal hyperplasia; 2) Cushing's syndrome; 3) galactorrhea;4) androgen-secreting tumors; 5) ethanol consumption>20g/day; 6) history of liver cirrhosis or other liver disease (viral hepatitis, autoimmune hepatitis, primary sclerosing cholangitis, primary biliary cirrhosis, drug-induced liver disease, hemochromatosis, Wilson's disease, α1-antitrypsin deficiency); 7) type 1diabetes mellitus; 8) uncontrolled hypothyroidism or hyperthyroidism; 9) adrenal insufficiency; 10) renal failure; 11) Malignant neoplasm; 12) pregnancy; 13) pre mature ovarian failure; 13) Known history of Drug addiction; 14) Smoking	No	No
**[24] Layegh 2016**	We designed this study to compare the existence of insulin resistance and its endocrinemetabolic consequences in obese and nonobese PCOS patients.	PCOS patients, age from 16-45 years were enrolled.	Patients with hyperprolactinemia, nonclassic CAH, Cushing syndrome, acromegaly, hypothyroidism, ovarian failure, adrenal and ovarian neoplasm and simple obesity were excluded based on history, clinical examination and appropriate laboratory tests wherever required. Patients having used any type of medication in previous three months and patients with known diabetes mellitus were also excluded.	NR	NR
**[25] Macut 2016**	The aim of the present study was	The study included all women who were	In all patients, non-classical 21-hydroxylase deficiency,	No	No

	to compare the prevalence of NAFLD between patients with PCOS and body mass index (BMI)-matched controls and to identify factors associated with the presence of NAFLD in PCOS.	consecutively diagnosed with PCOS and 125 BMI-matched healthy, control women between May 2008 and May 2013	hyperprolactinemia, Cushing's disease, untreated hypothyroidism and androgen secreting tumors were excluded prior to examination. In both patients and controls, alcohol consumption of .20 g/day and liver disease including viral, autoimmune, genetic and drug-induced were excluded. No patients or controls had received any medications and hormone treatment for at least 3 months before the study.		
**[28] C Jie 2017**	The aim of the present study was to investigate the relationship between hyperandrogenism and NAFLD in PCOS patients with body mass index (BMI), age and waist circumference matched controls and to identify factors associated with the presence of NAFLD in PCOS	Women with PCOS based on the revised Rotterdam 2003 criteria	In both patients and controls, alcohol consumption and liver disease, including viral, autoimmune, genetic, and drug-induced disease, were excluded	No	No
**[23] Kim 2017**	The aim of this study was to compare the prevalence of NAFLD in non-obese women with or without PCOS, and to determine the independent association between PCOS and NAFLD in a non-obese population.	This case–control study prospectively enrolled women with or without PCOS	We excluded subjects with excessive alcohol consumption (> 20 g/day measured by detailed questionnaire including alcohol amount and frequency),25 viral hepatitis (hepatitis B or C) or other chronic liver diseases.	No	No
**[30] Mehrabian 2017**	The first aim of the present study was to assess the prevalence of NAFLD in a sample of Iranian women with PCOS. The second aim was to define clinical predictive factors to determine PCOS women who should or should not be screened for insulin sensitivity and liver enzyme and liver ultrasonography.	Women with PCOS were included if they were between 18 and 42 years of age.	Other causes of irregular menstrual cycles, pregnancy, presence of viral hepatitis, hemochromatosis, liver disease, and other autoimmune disorders	No	No

**[34] Petta 2017**	In this view, in a large cohort of PCOS patients and of age-matched controls, we assessed whether PCOS firstly represents a risk factor for steatosis, and secondly whether insulin resistance and hyperandrogenism exerts a specific role in determining steatosis and fibrosis in affected women.	White women with PCOS	The following subjects were excluded from the study: women treated with clomiphene citrate, oral contraceptives, antiandrogens, drugs to control their appetite or insulin-sensitizing drugs during the 6 months before the examination; women with hyperprolactinemia; patients with basal 17-OH progesterone levels >6.05 nmol/l and peak >30.26 nmol/l at 60min after 250mg Synacthen; women with DHEAS >16.32 mmol/l who presented adrenal hyperplasia or adenoma or virilizing androgen-secreting neoplasias; and women whose clinical and hormone evaluation suggested Cushing's syndrome. We also excluded from the analysis women with evidence of viral infection (anti-HCV, anti-HIV, and HBsAg negativity) and a previous history of excessive alcohol consumption.	No	No
**[38] Sarkar 2018**	In the current study we aim to determine if ethnic differences in NAFLD/NASH are apparent in a population of women with PCOS	Prospective cohort comprised of women ages 18-45 years seen in a tertiary-referral PCOS clinic	Alternative diagnoses, including late-onset congenital adrenal hyperplasia, hypothyroidism, hyperprolactinemia, and functional hypothalamic amenorrhea were excluded by clinical and laboratory evaluation.Women with heavy alcohol use defined as > 7 drinks per week at the time of study enrollment were excluded. No women in the cohort had known chronic viral hepatitis.	No	No
**[45] Vassilatou 2018**	In this study we aimed to 1) evaluate the ability of Visceral adiposity index to identify the presence of hepatic steatosis and to 2) compare diagnostic performance of VAI to the one of three other indices of hepatic steatosis FLI, LAP and HSI in a cohort of premenopausal	We studies causasian premenopausal women aged 18-45 years with PCOS and BMI-matched healthy controls. Inclusion criteria for all women were 1) maximum alcohol consumption 1 alcoholic drink per day, or less 2) no history of known autoimmune or genetic or other chronic liver disease 3) absence of viral liver disease by appropriate tests and 4)	Described in inclusion criteria	No	No

	women with and without polycystic ovary syndrome (PCOS) assessed for NAFLD by ultrasonography.	absence of known hypertension, hyperlipidemia, diabetes mellitus or other systemic diseases. No use of any medication for at least 3 months was required prior to participation in the study.			
**[46] Zhang 2018**	In this study, the risk factors for PCOS complicated with NAFLD were analyzed, so as to provide clinical references for the early diagnosis and treatment of PCOS patients complicated with NAFLD.	PCOS patients based on the 2003 Rotterdam criteria	Exclusion criteria: i) patients complicated with other endocrine diseases (Cushing syndrome, thyroid dysfunction and premature ovarian failure); ii) patients with smoking history, alcohol abuse history, hypertension or coronary heart disease history; iii) patients with history of diseases in important organs, such as heart, liver or kidney; iv) patients who received drug therapy for PCOS in the past 3 months or took drugs affecting the glucose and lipid metabolism (such as glucocorticoids); v) patients with history of chronic liver disease (viral hepatitis, alcoholic hepatitis or other liver diseases).	No	No
**[41] Tantanavipas 2019**	The present study aimed to examine two parameters: (1) identify the predictive factors of NAFLD by US in PCOS and healthy women and (2) correlate and compare diagnostic accuracy for NAFLD between US and TE-based CAP.	Women with PCOS based on the Rotterdam criteria and healthy women with ages ranging from 20 to 40 years.	All participants were excluded if they had other related diseases, such as thyroid dysfunction, hyperprolactinemia, androgen-producing tumor, and Cushing's syndrome in addition to others. The other exclusion criteria included alcohol consumption >20 g/day, presence of known liver disease, and use of steatogenic medication or hormones for at least three months before the study.	No	No
**[27] Chakraborty 2020**	This study was aimed to evaluate the presence of NAFLD among adolescent women with PCOS by USG and evaluate the utility of non-invasive technique of TE on fibroscan as a diagnostic modality for PCOS.	Adolescent girls and young women in the age group of 16-24 yr attending endocrine clinic for complaints suggestive of PCOS were the potential individuals for the study. Those who qualified Rotterdam 2003 criteria for the diagnosis of PCOS were enrolled in the study. A control group included age-matched	The exclusion criteria were thyroid dysfunction, hyperprolactinaemia, Cushing's syndrome, non-classical congenital adrenal hyperplasia and androgen-secreting tumours. Women taking any drug(s) or hormonal preparations (e.g., metformin, oral contraceptive pills, anti-epileptics, etc.) that may influence weight or hepatic fat accumulation or those with known diabetes	No	No

		apparently healthy, non-hirsute woman who had long-term, predictable eumenorrhoea, without any history of endocrine disorders and normal ovarian morphology on USG.	mellitus (DM), renal, hepatic or cardiac dysfunction, were excluded.		
**[33] Oliveira de Lima 2020**	The current study was conducted to determine the prevalence, associated factors, and noninvasive fibrosis staging of NAFLD in a population of patients with PCOS at a single Brazilian center	Nonpregnant women with a diagnosis of PCOS	The subjects were excluded if they had a history of alcohol intake greater than 20 g per day or other chronic liver disease, or if using drugs that could cause liver steatosis.	No	No
**[37] Salva-Pastor 2020**	The objective of this studywas to determine the frequency of NAFLD development and sever-ity in Mexican patients with PCOS and matched-controls by age andbody mass index (BMI).	Mexican women with PCOS	Those patients who had established any of the followingdiagnoses were excluded from the study: hyperprolactinemia,pregnancy, dyslipidemia, thyroid or adrenal function alterations,diabetes mellitus, adrenal hyperplasia, Cushing syndrome, activeor latent viral infection hepatitis C, hepatitis B virus, or humanimmunodeficiency. Similarly, women who were in pharmaco-logical management at the time of the study or in the threemonths before the study, with hormonal contraceptives, anti-androgens, insulin receptor sensitizers, glucagon-like peptideanalogs, and clomiphene citrate or infertility treatment protocol.Finally, patients with known chronic liver disease or significantalcohol consumption defined as > 7 drinks per week were not included	No	No
**[39] Shengir 2020**	We employed TE with CAP in consecutive PCOS patients from South Asia as a part of a routine screening program with the following aims: (1) To assess prevalence and associated	We included patients with PCOS defined by the modified Rotterdam criteria, after excluding other endocrine disorders. All patients met at least two criteria among clinical (hirsutism and/or other signs and symptoms of hyperandrogenism, i.e.,	Exclusion criteria were the following: (1) Positivity for hepatitis C virus antibody or hepatitis B virus (HBV) surface antigen; (2) History of pre-existing liver disease or new diagnosis at the screening visit (auto-immune hepatitis, primary biliary cholangitis, primary sclerosing cholangitis,	No	No

	predictors of NAFLD; (2) To determine prevalence of significant liver fibrosis.	acne/seborrhea and alopecia) and/or biochemical hyperandrogenism, ovulatory dysfunction and polycystic ovarian morphology	hemochromatosis, Wilson's disease, alpha-1 anti-trypsin); (3) History of hepatocellular carcinoma, liver transplantation or decompensated liver disease (ascites, hepatorenal syndrome, spontaneous bacterial peritonitis, hepatic encephalopathy, variceal hemorrhage); (4) Hazardous alcohol intake, as estimated by an Alcohol Use Disorders Identification Test (AUDIT-C) score ≥ 7[21]; (5) Pregnancy at time of recruitment; and (6)Failure of TE examination or unreliable measurement		
**[47] Zheng 2020**	In the present study, we aimed to explore whether the new adiposity indices (LAP, VAI, and TyG) could predict the risk of HS quantified by CAP in women with PCOS. We also aimed to evaluate whether the new adiposity indices are better than the traditional anthropometric parameters (WC and BMI) to predict the risk of HS.	Women aged 20 to 40 years with a PCOS diagnosis	All participants were excluded if they had other related diseases, such as thyroid dysfunction, late-onset congenital adrenal hyperplasia, or androgensecreting tumors. The other exclusion criteria included alcohol consumption >20 g/day, presence of known liver disease such as viral or autoimmune hepatitis, viral hepatitis, and treatment with hepatotoxic medications.	No	No
**[31] Bin Won 2021**	This study was conducted to evaluate risk factors associated with NAFLD occurrence to determine better identifiers in screening for metabolic abnormalities at the time of PCOS diagnosis and consider its underlying cause.	586 women diagnosed with PCOS aged 13–35 years from January 2010 to April 2018 at the Department of Obstetrics and Gynecology in Severance Hospital, Seoul, Korea, were evaluated. Patients were included if they were diagnosed with PCOS. NAFLD was defined as simple fatty liver, nonalcoholic steatohepatitis, and/or liver fibrosis23 after excluding other—viral, alcoholic, iatrogenic by medication causes of liver diseases.	Patients were excluded from the analysis if they had any of following exclusion criteria: other causes of irregular menstrual cycles or androgen excess including hyperprolactinemia, uncontrolled thyroid disease, congenital adrenal hyperplasia, Cushing's disease, androgen secreting tumor, or pregnancy; who were diagnosed with PCOS at a department apart from the Department of Obstetrics and Gynecology; anyone with health conditions that could influence liver function; a history of hormonal contraception or metformin use within 3 months preceding the diagnosis of PCOS or NAFLD; and those with inadequate data or no	No	No

			follow-up		
**[40] Siwatch 2021**	In the present study, we sought to know the prevalence and characteristics of NAFLD in Indian PCOS women.	Patients attending infertility clinic in the department of gynaecology and found to have PCOS according to Rotterdam criteria (Rotterdam 2004) were enrolled in the study. Seventy age- and body mass index (BMI)-matched women without evidence of PCOS attending the infertility clinic, were taken as the controls. The inclusion criteria for cases included women who were (1) between ages 18 and 40 years, (2) with PCOS diagnosed by Rotterdam or NIH criteria (Rizzo et al. 2009), (3) not on any treatment for PCOS or therapy that may influence metabolic or hepatic biochemical parameters in the past three months and (4) willing to participate in the study.	The exclusion criteria included: (1) women with other reasons of irregular menstruation or androgen excess including hyperprolactinaemia, uncontrolled thyroid disease, androgen secreting tumours, congenital adrenal hyperplasia, Cushing's syndrome, (2) history of known liver disease or other medical issues that could cause transaminitis, (3) history of medication use known to raise the levels of liver enzymes like NSAIDS, anti-epileptics, antibiotics, erythromycin, tetracycline, amoxyclav, isoniazid and statins, etc. or (4) a history of alcohol consumption >14 standard drinks on average per week	No	No
**[20] Arikan 2022**	The present study used liver ultrasound to investigate the predictivity of fatty liver index for NAFLD diagnoses in lean and overweight/obese PCOS patients	The study population was composed of females admitted at the Namik Kemal University Faculty of Medicine Department of Obstetrics and Gynecology/Premenopausal volunteer women, aged between 18-47 years were included	We excluded patients who had received treatment for PCOS within the prevous six months, pregnant women, those who consumed more than 20g alcohol daily, those who had used oral contraceptives within the previous three months, and those with any of the following diagnoses: hypothyroidism, hyperthyroidism, hyperprolactinemia, diabetes mellitus, Cushing syndrome, congenital adrenal hyperplasia, androgen-secreting ovarian or adrenal tumor, chronic viral hepatitis, hemochromatosis and autoimmune hepatitis, neoplastic, metabolis, or cardiovascular diseases, and history of chronic diseases.	No	No

NR: not reported

4. Table S2. Definitions and cut-off values used to diagnose NAFLD

Author/y	Author/y	NAFLD assessment method	NAFLD definition
**[51] Cerda 2007**	**Cerda 2007**	Ultrasound	The presence of fatty liver was determined in a qualitative manner using accepted criteria including a bright hepatic echo pattern (compared with the right kidney), a homogeneous or coarse echo pattern, increased attenuation of the US beam, and loss of intrahepatic architectural details
**[54] Gambarin-Gelwan 2007**	**Gambarin-Gelwan 2007**	Ultrasound	The severity of hepatic steatosis was graded as absent, mild, moderate, or severe on the basis of the echogenicity of the liver parenchyma and the visualization of intrahepatic vessels and the diaphragm
**[26] Markou 2010**	**Markou 2010**	CT-Scan	Fatty infiltration of the liver was considered when the CT-attenuation of the liver was at least 10 Housefield units below the CT attenuation of the spleen
**[29] Ma 2011**	**Ma 2011**	Ultrasound	Fatty liver was diagnosed during the same ultrasound examination according to the criteria of Scatarige et al. These criteria included an increase in hepatic echogenicity, decreased penetration of the deep part of the liver, and decreased echogenicity of the diaphragm and intrahepatic portal vessels.
**[19] Eldesoky 2012**	**Eldesoky 2012**	Ultrasound	Fatty liver was determined by abdominal ultrasound using standardized criteria in a qualitative manner using accepted criteria including a bright hepatic echo pattern (compared with the right kidney), a homogeneous or coarse echo pattern, increased attenuation of the ultrasound beam, and loss of intrahepatic architectural details
**[22] Karoli 2012**	**Karoli 2012**	Ultrasound	Heapatic steatosis was defined as diffuse increase in fine echoes in liver parenchyma with impaired visualization of intrahepatic vessels and the diaphragm
**[48] Zueff 2012**	**Zueff 2012**	Ultrasound	Diagnosis and stage of NAFLD were assessed as follows: Grade I (mild), increased echogenicity of liver compared with renal cortex or spleen; Grade II (moderate), obscured hepatic and portal vein walls; Grade III (severe), impaired visibility of the diaphragm
**[49] Borruel 2013**	**Borruel 2013**	Ultrasound	Hepatic steatosis was diagnosed when the echogenicity of the liver was higher than the echogenicity of the right kidney and was graded as follows: grade 0, normal echogenicity; grade 1, slight, diffuse increase in fine echoes in liver parenchyma with normal visualization of the diaphragm and intrahepatic vessel borders; grade 2, moderate, diffuse increase in fine echoes with slightly impaired visualization of the intrahepatic vessels and diaphragm; and grade 3, marked increase in fine echoes with poor or nonvisualization of the intrahepatic vessel borders, diaphragm, and posterior right lobe of the liver.
**[32] Michaliszyn 2013**	**Michaliszyn 2013**	CT-Scan	Fatty liver was diagnosed by an index represented as a ratio between liver to spleen Hounsfield attenuation units (HU)

			(liver HU/spleen HU). Fatty liver index <1.0 was indicative of fatty liver and correlated with hepatic fat volume percent
**[35] Qu 2013**	**Qu 2013**	Ultrasound	NAFLD severity was graded as mild, moderate, and severe based on echogenicity of the liver parenchyma and the visualization of intrahepatic vessels and the diaphragm
**[43] Tarantino 2013**	**Tarantino 2013**	Ultrasound	The classification of bright liver or hepatis steatosis severity was based on the following scale of hyperechogenity: o= absent, 1=light, 2= moderate, 3=severe, pointing out the difference between the densities of the liver
**[42] Bohdanowicz-Pawlak 2014**	**Bohdanowicz-Pawlak 2014**	Ultrasound	The degree of fatty infiltration of the liver was classified as mild (hepatic steatosis grade 1), moderate (hepatic steatosis grade 2), or severe (hepatic steatosis grade 3).
**[21] Kahal 2014**	**Kahal 2014**	Ultrasound	NR
**[44] Tock 2014**	**Tock 2014**	Ultrasound	Hepatic steatosis was defined according to the criteria of Scatarige et al. and included increased hepatic echogenicity, posterior acoustic attenuation of the liver, and reduced echogenicity of the intrahepatic portal vein. Patients were classified according to the absence or presence of liver fat as well as the degree of fatty infiltration: grade 1 (mild steatosis), grade 2 (moderate steatosis), and grade 3 (severe steatosis).
**[50] Çağlar 2015**	**Çağlar 2015**	Ultrasound	Fatty liver was diagnosed by abdominal ultrasound using accepted criteria which includes a bright hepatic echo pattern, increased attenuation of the ultrasound beam, and loss of intrahepatic architectural details
**[36] Romanowski 2015**	**Romanowski 2015**	Ultrasound	For the screening for hepatic steatosis, hepatic parenchymal echo texture was evaluated and compared with the echo texture of the spleen. When isoechogenic, the liver parenchyma is considered normal, that is, without evidence of steatosis. The presence of hyperechoic hepatic parenchyma (bright liver) is considered a characteristic of hepatic steatosis
**[52] Cree-Green 2016**	**Cree-Green 2016**	MRI	>5% of liver fat on MRI
**[53] El-Tahawy 2016**	**El-Tahawy 2016**	Ultrasound	For the screening for hepatic steatosis, hepatic parenchymal echo texture was evaluated and compared with the echo texture of the spleen. When isoechogenic, the liver parenchyma is considered normal, that is, without evidence of steatosis. The presence of hyperechoic hepatic parenchyma (bright liver) is considered a characteristic of hepatic steatosis
**[24] Layegh 2016**	**Layegh 2016**	Ultrasound	NR
**[25] Macut 2016**	**Macut 2016**	NAFLD fatty liver fat score	The NAFLD liver fat score (NAFLD-LFS) value of >-0.640 was considered diagnostic of NAFLD
**[28] C Jie 2017**	**C Jie 2017**	Ultrasound	The quantitative measurement of LFC was performed by ultrasonography. The LFC

			was calculated according to the following predictive formula: liver fat content (%) = 62.592 * standardized ultrasound hepatic/renal ratio + 168.076 * standardized hepatic attenuation rate – 27.863.
**[23] Kim 2017**	**Kim 2017**	Ultrasound	Fatty liver was diagnosed if the echogenicity of the liver was higher than the echogenicity of the right kidney
**[30] Mehrabian 2017**	**Mehrabian 2017**	Ultrasound	The presence of fatty liver was determined in a qualitative manner using accepted criteria including a bright hepatic echo pattern, (compared with the right kidney) a homogeneous or coarse echo pattern, increased attenuation of the US beam, and loss of intrahepatic architectural details
**[34] Petta 2017**	**Petta 2017**	Hepatic steatosis index	Steatosis was diagnosed by using the validated hepatic steatosis index (HSI) that takes into account the following parameters: BMI, AST/ALT, gender, diabetes. The score was calculatedusing the original reported formula, and patients where considered with steatosis if HSI>36.
**[38] Sarkar 2018**	**Sakar 2018**	Transient elastography	CAP ≥ 250 dB/m
**[45] Vassilatou 2018**	**Vassilatou 2018**	Ultrasound	Detection of HS was based on the assessment of the following ultrasonographic parameters, as previously described [21]: ultrasonographic contrast between hepatic and right renal parenchyma (hepatorenal echo contrast), abnormally intense high-level echoes arising from the hepatic parenchyma, echo penetration into the deep portion of the liver, intrahepatic vessel blurring and abnormal visualization of the diaphragm. Absence of HS was defined as equal echogenicity of hepatic parenchyma to that of the renal cortex with clear visualization of the intrahepatic vessels and the diaphragm
**[46] Zhang 2018**	**Zhang 2018**	Ultrasound	NR
**[41] Tantanavipas 2019**	**Tantanavipas 2019**	Ultrasound	Diagnosis was made with ultrasound. US measured the extent of brightness or diffusely increased echogenicity of the liver parenchyma, echogenic discrepancy of the liver and the kidney, and loss echogenicity of portal venous walls
**[27] Chakraborty 2020**	**Chakraborty 2020**	Ultrasound	Fatty liver on ultrasound was quantitated as grade I: increased hepatic echogenicity with visible periportal and diaphragmatic echogenicity, grade II: increased hepatic echogenicity with imperceptible periportal echogenicity, without obscuration of the diaphragm, grade III: increased hepatic echogenicity with imperceptible periportal echogenicity and obscuration of the diaphragm.
**[33] Oliveira de Lima 2020**	**Oliveira de Lima 2020**	Ultrasound	Fatty liver was determined in the presence of a higher echogenicity in the hepatic parenchyma compared with the renal cortex and with impaired visualization of the intrahepatic vessels and diaphragm
**[37] Salva-**	**Salva-Pastor 2020**	Transient elastography	CAP≥232 dB/m

**Pastor 2020**			
**[39] Shengir 2020**	**Shengir 2020**	Transient elastography	CAP≥288 dB/m
**[47] Zheng 2020**	**Zheng 2020**	Transient elastography	CAP≥248 dB/m
**[31] Bin Won 2021**	**Bin Won 2021**	Ultrasound/fibroscan/MRI	CAP≥238 dB/m
**[40] Siwatch 2021**	**Siwatch 2021**	Ultrasound	An abdominal ultrasound was done to assess fatty liver. Normal hepatic parenchyma, i.e. higher or equal to that of the renal cortex, with clearly seen intrahepatic vessels and diaphragm, signified no steatosis. Mild hepatic steatosis was defined by slight diffuse increased echogenicity of the hepatic parenchyma with clear visualisation of the intrahepatic vessels and the diaphragm. A moderate diffuse increase in fine echoes in the hepatic parenchyma which slightly impaired visualisation of the intrahepatic vessels/the diaphragm signified moderate hepatic steatosis while severe steatosis was seen as a marked increase in fine echoes in the liver with poor visualisation of intrahepatic vessels/the diaphragm and the posterior right hepatic lobe
**[20] Arikan 2022**	**Arikan 2022**	Ultrasound	Grade 1 hepatic steatosis was defined as the presence of bright echoes or increased hepatorenal contrast, Grade 2 hepatic steatosis as the presence of both bright echoes and increased hepatorenal contrast, as well as vessel blurring and Grade 3 steatosis, was considered to be present when in addition to the criteria for Grade 2, there was evidence of posterior beam attenuation and non-visualization of the diaphragm

5. META-ANALYSIS OF OBSERVATIONAL STUDIES IN EPIDEMIOLOGY (MOOSE) CHECKLIST

Criteria		Brief description of how the criteria were handled in the meta-analysis
**Reporting of background should include**		
√	Problem definition	Polycystic ovary syndrome (PCOS) is characterized by hyperandrogenism, ovulatory dysfunction, and polycystic ovarian morphology [3]. It is closely linked with obesity, insulin resistance, abnormal glucose metabolism, dyslipidemia, and related disorders, which increase the chance of metabolic-associated abnormalities, including NAFLD. Indeed, previous studies have demonstrated an association between PCOS and NAFLD [4–6], and it has

		been postulated that the metabolic environment of PCOS patients favors the build-up of fat in the liver. Therefore, it is paramount to determine the burden of NAFLD in patients affected by PCOS and further identify the factors mediating the relationship between NAFLD and PCOS. Understanding the link between the two disorders will advance the field of NAFLD in special populations and aid stakeholders and decision-makers in designing and implementing actions to tackle the NAFLD burden, focusing on groups at higher risk of the condition.<br><br>We performed a systematic review (SR) and meta-analysis (MA) to assess NAFLD's prevalence and risk factors in patients with PCOS.
√	Hypothesis statement	Not reported
√	Description of study outcomes	**Outcomes**<br><br>The prevalence of NAFLD in PCOS patients and the factors associated (risk factors) with such prevalence were the outcomes of interest in this systematic review.<br><br>NAFLD diagnosis was based on the detection of hepatic steatosis, defined as the presence of significant steatosis demonstrated either by biopsy or a non-invasive test. For this SR, biopsy, non-invasive imaging tests, and blood biomarkers/panels were considered appropriate diagnostic methods and thereby eligible for inclusion 10.<br><br>Non-invasive imaging tests included right upper quadrant ultrasound, computer tomographic (CT) scan, magnetic resonance imaging (MRI) techniques, or vibration-controlled transient elastography (VCTE)-based attenuation parameter (CAP) measurements. Non-invasive blood biomarkers and panels are described elsewhere 11. If studies reported the assessment of liver steatosis by any of the methods mentioned above with their correspondent definition, then the study was considered to inform the primary outcome.<br><br>Regarding the risk factors for NAFLD in PCOS patients, we were interested in adjusted measures of association between PCOS and NAFLD. Therefore, reported measures relating to patient characteristics, relevant clinical data, and comorbidities were extracted from the

		included studies. The extracted risk factors had to result from a multivariable regression analysis performed in a PCOS population with NAFLD as the outcome variable and reporting adjusted odds ratios with 95% confidence intervals for the covariates of interest. We did not pre-specify risk factors of interest; instead, we extracted those available in the included studies. We collected the risk factors (adjusted odds ratios from a multivariable regression analysis) that were available in the included articles. If two studies or more reported the same factor, we considered it appropriate for meta-analysis.
√	Type of exposure or intervention used	**Participants**<br><br>The participants were women with PCOS diagnosed by standard criteria 3. Articles were included in this study if the diagnosis of PCOS was performed by any of the following: 1) National Institutes of Health criteria, 2) Rotterdam criteria, or 3) the Androgen Excess and PCOS Society criteria. These criteria share several diagnostic elements, including the combination of hyperandrogenism, ovulatory dysfunction, and polycystic ovarian morphologic features 3. Cochrane guidelines 8 advise that reviews should be sufficiently broad to encompass the likely diversity of studies, and inclusion criteria should aim to include all relevant clinical features with which patients of interest would present. Therefore, we did not restrict the participant's inclusion criteria to a specific set of diagnostic criteria because all of them reflect the clinical problem of interest (PCOS). Excluding studies based on the diagnostic criteria used to diagnose PCOS may result in exclusion of informative studies containing clinically relevant data from patients with the clinical problem of interest.
√	Type of study designs used	**Types of studies**<br><br>We included observational studies, including cohort, case-control studies, and case series. We also considered randomized controlled trials. These studies were included if the proportion of patients with NAFLD among PCOS patients was assessed. Articles that, in addition, assessed the factors associated with NAFLD were also considered eligible. Case reports were not considered eligible for inclusion.

√	Study population	**Participants**<br><br>The participants were women with PCOS diagnosed by standard criteria 3. Articles were included in this study if the diagnosis of PCOS was performed by any of the following: 1) National Institutes of Health criteria, 2) Rotterdam criteria, or 3) the Androgen Excess and PCOS Society criteria. These criteria share several diagnostic elements, including the combination of hyperandrogenism, ovulatory dysfunction, and polycystic ovarian morphologic features 3. Cochrane guidelines 8 advise that reviews should be sufficiently broad to encompass the likely diversity of studies, and inclusion criteria should aim to include all relevant clinical features with which patients of interest would present. Therefore, we did not restrict the participant's inclusion criteria to a specific set of diagnostic criteria because all of them reflect the clinical problem of interest (PCOS). Excluding studies based on the diagnostic criteria used to diagnose PCOS may result in exclusion of informative studies containing clinically relevant data from patients with the clinical problem of interest.
**Reporting of search strategy should include**		
√	Qualifications of searchers	Two authors (RM, MSD) independently screened the titles and abstracts and selected potential articles for inclusion based on the inclusion and exclusion criteria. Finally, articles that appeared relevant to this SR's topic were retrieved as full text and subsequently reviewed by two investigators (RM, MSD) who independently applied inclusion and exclusion criteria to full texts for final eligibility.
√	Search strategy, including time period included in the synthesis and keywords	Following experts' recommendations 12,13, we outlined a systematic search strategy of the available literature. The literature search was performed from inception to June 2022 in SCOPUS, MEDLINE (through Ovid), and Scielo. The searches were not restricted to language or geographic location. The systematic database searching was complemented by a snowball scanning of the references cited in the included studies. The structure of the search strategies in the electronic databases was informed by the main concepts of the review, combining

		controlled vocabulary and synonyms related to the population of interest (patients with PCOS) and the diease/condition of interest (NAFLD). The search strategies that were executed in SCOPUS, MEDLINE and Scielo are described in detail in the supplementary file.
√	Databases and registries searched	SCOPUS, MEDLINE and Scielo
√	Search software used, name and version, including special features	We did not employ a search software. Rayyan was used to handle the process of abstract screening and duplicates removal.
√	Use of hand searching	We hand-searched references of retrieved papers for additional references
√	List of citations located and those excluded, including justifications	Figure 1. PRISMA DIAGRAM
√	Method of addressing articles published in languages other than English	We placed no restrictions on language. We were able to obtained all articles potentially eligible for inclusion in English language
√	Method of handling abstracts and unpublished studies	We did not include unpublished or abstracts- We included only publications
√	Description of any contact with authors	When needed, we intended to contact the original anthor for clarification. This was not requiered
**Reporting of methods should include**		
√	Description of relevance or appropriateness of studies assembled for assessing the hypothesis to be tested	Detailed inclusion and exclusion criteria are described in the paper
√	Rationale for the selection and coding of data	We collected the data of interest from each study into a pre-designed data collection form. The data was collected as reported in each study and included: authors, year of publication, study design, region of origin, number of patients (in comparative studies: number of PCOS and control patients), comorbidity information, and relevant lab values. We extracted and charted data only from PCOS patients. In the case of comparatives studies, the control group's data were not considered nor extracted.

		We also extracted each study's objectives and inclusion/exclusion criteria. This information was extracted and collected in the same form as reported in each study and is available in Table 1S in the Supplementary file.
√	Assessment of confounding	To explore the heterogeneity of the pooled prevalence obtained from the proportion meta-analysis, we performed subgroup analyses stratified by study design, geographical location, and NAFLD and PCOS diagnostic methods/criteria.<br><br>Using the effect estimates (estimated prevalence) and its standard errors obtained from the proportion meta-analysis, we performed a meta-regression analysis to explore if selected study-level summary data could influence the estimated prevalence.
√	Assessment of study quality, including blinding of quality assessors; stratification or regression on possible predictors of study results	We used the Methodological Index for Non-Randomized Studies (MINORS) 15 tool to assess the quality and internal validity of the studies included in this SR. The explanation of how to use it is described elsewhere 15. MINORS evaluates the methodological quality of non-randomized studies across eight methodological items in cases of observational studies without a comparison group. Four additional items are added in the case of a comparative study. Each item is scored as 0: if not reported (Red: high risk of bias); 1: reported but inadequate (Yellow: unclear risk of bias); and 2: reported and adequate (Green: low risk of bias). The results from the MINORS evaluation are available in the Supplementary file. Publication bias was not evaluated because tests to evaluate this kind of bias were created to be performed in cases of comparative data. However, there is no evidence that these tests are appropriate for meta-analyses of proportions. On the contrary, conventional methods to assess publication bias are inaccurate in this context 16, and experts recommend against performing statistical calculations to assess it when conducting meta-analyses of proportions 17.
√	Assessment of heterogeneity	We used the I^2 value to assess heterogeneity
√	Description of statistical methods in sufficient detail to	The "metaprop_one" command is appropriate to pool proportions in a meta-analysis of proportions to estimate

	be replicated	the prevalence of an event of interest such as in this SR. In brief, confidence intervals for the individual studies were calculated using exact confidence limits for a binomial proportion. Pooled event rates (pooled prevalence) were estimated through a meta-analysis of binomial data with the Freeman-Tukey double arcsine transformation of proportions 18. The results from this analysis were presented in a forrest plot presenting the study-specific proportions with 95% confidence intervals the I2 statistic and the overall pooled estimate (the estimated NAFLD prevalence).<br><br>Risk factors of NAFLD among patients with PCOS were meta-analyzed when possible. When at least two studies reported the same factor with its corresponding odds ratio from a multivariable regression analysis, we combined such effect estimates (adjusted odds ratios) to produce a pooled OR in a meta-analysis. To meta-analyze these precalculated effect estimates, we used the log-odds ratios with their 95% confidence intervals as inputs to the analysis; however, the results of these meta-analyses are presented on the ratio scale (adjusted ORs with 95% CI).<br><br>In both meta-analyses (proportions and pre-calculated effect sizes), heterogeneity was evaluated using the I2 test, corresponding to low (I2<25%), medium (I2=25-75%), and high (I2>75%) heterogeneity.<br><br>**Subgroup analyses and meta-regression**<br><br>To explore the heterogeneity of the pooled prevalence obtained from the proportion meta-analysis, we performed subgroup analyses stratified by study design, geographical location, and NAFLD and PCOS diagnostic methods/criteria.<br><br>Using the effect estimates (estimated prevalence) and its standard errors obtained from the proportion meta-analysis, we performed a meta-regression analysis to explore if selected study-level summary data could influence the estimated prevalence.<br><br>We extracted the prevalence of metabolic syndrome and the mean values of HOMA-IR, free androgen index, and

		total testosterone (nmol/L) from the studies where they were available. We assumed that any effect on the estimated prevalence was mediated through changes in the variables of interest. Therefore, we performed a random-effects meta-regression to estimate the coefficients, β, which indicate how the estimated prevalence changed with a unit increase in the proposed explanatory variables. A REML algorithm estimated the between-study variance in this model. The results of the meta-regression were presented in "Bubble plots" with fitted meta-regression lines, with circles representing the estimates from each study, sized according to each estimate's precision.<br><br>All statistical analyses were performed in Stata statistical software v.14.
√	Provision of appropriate tables and graphics	See tables included in the paper. See forest plot of the pooled prevalence and graphics illustrating the results of meta-regression.
**Reporting of results should include**		
√	Graph summarizing individual study estimates and overall estimate	Figure 2
√	Table giving descriptive information for each study included	Table 1
√	Results of sensitivity testing	Table 2
√	Indication of statistical uncertainty of findings	Pooled proportion (prevalence), 95% CI and I^2
**Reporting of discussion should include**		
√	Quantitative assessment of bias	See methods and results
√	Justification for exclusion	See Methodology section and PRISMA diagram
√	Assessment of quality of included studies	Supplementary File.

**Reporting of conclusions should include**		
√	Consideration of alternative explanations for observed results	Discussion section.
√	Generalization of the conclusions	Discussion section
√	Guidelines for future research	Discussion section
√	Disclosure of funding source	See the manuscript
